# Psychosocial Interventions for Depressive and Anxiety Symptoms in Individuals with Chronic Kidney Disease: Systematic Review and Meta-Analysis

**DOI:** 10.3389/fpsyg.2017.00992

**Published:** 2017-06-13

**Authors:** Michaela C. Pascoe, David R. Thompson, David J. Castle, Samantha M. McEvedy, Chantal F. Ski

**Affiliations:** ^1^Department of Cancer Experience Research, Peter MacCallum Cancer CentreMelbourne, VIC, Australia; ^2^Department of Psychiatry, University of MelbourneMelbourne, VIC, Australia; ^3^Department of Epidemiology and Preventive Medicine, Monash UniversityMelbourne, VIC, Australia; ^4^Mental Health Service, St. Vincent's HospitalMelbourne, VIC, Australia; ^5^School of Psychology and Public Health, La Trobe UniversityMelbourne, VIC, Australia

**Keywords:** depression, anxiety, quality of life, psychosocial interventions, chronic kidney disease

## Abstract

**Purpose:** Depressive and anxiety symptoms are common amongst individuals with chronic kidney disease and are known to affect quality of life adversely. Psychosocial interventions have been shown to decrease depressive and anxiety symptoms in various chronic diseases, but few studies have examined their efficacy in people with chronic kidney disease and no meta-analysis has been published. Thus, the aim of the present systematic review and meta-analysis was to evaluate the effects of psychosocial interventions on depressive and anxiety symptoms as well as quality of life in individuals diagnosed with chronic kidney disease and/or their carers.

**Methods:** In this systematic review and meta-analysis, we included published randomized controlled trials comparing psychosocial interventions versus usual care for impacting depressive and anxiety symptoms and quality of life.

**Results:** Eight studies were included in the systematic review and six of these were subjected to meta-analysis. Psychosocial interventions were associated with a medium effect size for reduction in depressive symptoms and a small effect size for improved quality of life in the in individuals with chronic-kidney-disease and their carers. Some evidence suggested a reduction in anxiety.

**Conclusion:** Psychosocial interventions appear to reduce depressive symptoms and improve quality of life in patients with chronic-kidney-disease and their carers and to have some beneficial impact on anxiety. However, the small number of identified studies indicates a need for further research in this field.

## Introduction

Chronic kidney disease (CKD) is a progressive loss in kidney function characterized by the kidneys failure to clean toxins and waste products from the blood. The worldwide prevalence of CKD is 8–16% (Ene-Iordache et al., 2016). There are five stages of CKD, measured using a test of glomerular-filtration-rate (GFR), which estimates how much blood passes through the glomeruli each minute. A GFR of <15 ml/min is referred to as stage-5 of CKD, marking kidney failure and the need for dialysis; it is also termed end-stage-kidney-disease (ESKD) (Kidney Health Australia, 2016b). Approximately 500,000 individuals worldwide develop ESKD every year (Ojo, 2014). Accordingly, CKD is a global challenge (GBD Mortality Causes of Death Collaborators, 2015). The total cost associated with the treatment of CKD in Australia was AUD$4.1 billion in 2012 (Kidney Health Australia, 2016a), US$55 billion in the USA in 2010 (Honeycutt et al., 2013) and $1.45 billion in the UK in 2009–2010 (Kerr et al., 2012). Given the high prevalence and associated cost to the community, it is important to understand the factors that influence prognosis in order to achieve the best possible health outcomes.

Depressive and anxiety symptoms are important factors affecting prognostic outcome and quality of life (QoL) in individuals with CKD, including ESKD (Lee et al., 2013). Indeed, renal dialysis places a considerable burden on patients with CKD and often compromises their QoL, leading to high levels of anxiety and depression (Theofilou, 2011). Self-reports show that depressive symptoms and anxiety affect ~25% of individuals with CKD (Stasiak et al., 2014). Using Structured Clinical Interview, 71% of haemodialysis patients met the criteria for clinical anxiety according to the Diagnostic and Statistical Manual of Mental Disorders-IV (DSM-IV), in a sample of 70 individuals (Cukor et al., 2008). Structured Clinical Interview showed that the prevalence of a major depressive episode was 21% in a sample of 272 consecutive CKD participants and did not vary significantly among different CKD stages (Hedayati et al., 2009).

Depressive symptoms are associated with reduced treatment adherence, impaired functional capacity and higher rates of hospitalization (Hedayati et al., 2010). There is also an association with increased rates of withdrawal from dialysis and earlier mortality (Lacson et al., 2012). Despite this, there has been only limited research on interventions to prevent or manage depressive symptoms in CKD populations. There has been even less research into the association between anxiety and outcomes in this patient group. Very little rigorous research has investigated how to prevent or manage these issues effectively, though one study demonstrated that a nurse practitioner model of care was associated with improved QoL amongst ESKD patients receiving dialysis (Stanley et al., 2015).

Some studies have reported that psychosocial interventions, i.e., a combination of psychological [e.g., cognitive behavioral therapy (CBT)] and social (e.g., social support) components, decrease depression and anxiety in patients with coronary heart disease and depression (Subasinghe et al., 2015) and in stroke (Eldred and Sykes, 2008). However, there is a paucity of studies examining the role of psychosocial interventions in patients with CKD. Thus, with a view to determining whether there is scope to develop further research in this area, we conducted a systematic review, and where appropriate a meta-analysis, of studies examining the effects of psychosocial interventions on depressive symptoms, anxiety symptoms and QoL among individuals with CKD.

## Methods

This study was conducted following the Preferred Reporting Items for Systematic Reviews and Meta-Analyses (PRISMA) guidelines (Moher et al., 2010). A prospective protocol for the systematic review was not previously published.

### Criteria

Eligible studies were randomized controlled trials (RCTs) published in English that included: individuals or the carers of individuals diagnosed with CKD (including ESKD); evaluation of a psychosocial intervention; and outcomes of symptoms of depression, anxiety, or QoL. Dissertations which had not been published as scientific papers, were excluded. As we were interested in the effects of psychosocial interventions in adults, studies involving children/adolescents were excluded.

### Search strategy

Searches were undertaken in December-2015 and updated in May-2016 for title or MeSH words, “kidney-disease,” or “renal-disease,” or “renal-insufficiency,” or “dialysis,” or “peritoneal-dialysis,” or “hemodialysis,” or “haemodialysis,” or “kidney-function,” or “kidney-failure,” and the specific abstract words, “depression,” or “depressive,” “depressed,” or “melancholia,” or “dysthymia,” or “mood,” or “anxiety,” or “anxious,” or “quality-of-life,” or “coping,” or “stress,” and the specific abstract word, “psych^*^,” or “motivational-interviewing,” or “motivational-behavior,” or “behavior-interviewing,” or “behavior-change,” or “motivational-behavior,” or “behavior-interviewing,” or “behavior-change,” or “motivational-change,” or “non-invasive-change. Articles were obtained by searches of the electronic databases, PubMed, MEDLINE, CINAHL, PsycINFO, Scopus and Web-of-Science, SocIndex, and the Cochrane-Central-Register-of-Controlled-Trials (Blackhall, 2007). Authors of eligible studies were contacted to request unpublished data, where applicable. In studies that included some non-CKD patients, only data relating to the patients with CKD was extracted/included in the analyses.

### Study selection

Sourced studies were imported into Covidence Online Software (https://www.covidence.org). Two independent reviewers screened studies for relevance based on titles/abstracts and later full-texts (MCP, SMM) with disagreements resolved through discussion or by consulting a third reviewer (CFS).

### Data extraction

Data were extracted using a predesigned form and included study design, country undertaken, aims, ethical information, studied outcomes, sample size, participant characteristics, and intervention characteristics. Means (*M*), standard deviations (*SD*), and sample sizes (*n*) were extracted. Study authors were contacted if published data were incomplete or unclear. Data were extracted independently by two reviewers (MCP, SMM) with no disagreements arising.

### Risk of bias in individual studies and grades of recommendation, assessment, development, and evaluation

Methodological quality of the included studies was assessed independently by two reviewers (MCP, SMM) using the Cochrane Collaboration's risk of bias assessment tool (Higgins and Green, 2011). Due to the nature of the studies reviewed, blinding of participants and personnel was not assessed as it is not possible to blind the person delivering or receiving the intervention or usual care (UC). To best capture the current state and quality of research in this field, papers were not included or excluded based on quality assessment, and thus all eligible articles were included. Grades of Recommendation, Assessment, Development and Evaluation (GRADE), was assessed using the GRADE working group recommendations as published in the Cochrane Handbook (Higgins and Green, 2011). We considered five factors when assessing the quality of evidence, namely: (1) risk of bias, (2) heterogeneity, (3) population, intervention, comparison, outcomes (PICO), (4) precision, and (5) publication bias (Higgins and Green, 2011).

### Summary measures

For the meta-analysis we report the standardized mean difference (*SMD*), where the mean difference in each study is divided by the *SD* to create an index that is comparable across studies (Borenstein et al., 2009). The *SMD* was used in place of mean difference as the studies included in the meta-analysis used different scales not comparable in raw form (Borenstein et al., 2009). The Hedges' G (*g*), form of the *SMD* was used. Where multiple outcomes were used to measure the depressive symptoms, anxiety symptoms, or QoL outcomes, composite scores using the mean of the relevant scales were used, as shown in **Table 4**. Using this validated method, the mean (*M*) and variance of the composite are computed by performing a fixed-effect meta-analysis on the study subgroups, the variance for the study is half as large as either subgroup since it is based on double as much information. This procedure forms a composite effect size and variance, which is then used in the meta-analysis (Borenstein et al., 2009).

We report the confidence interval (*CI*), the range in which the *SMD* could fall, the *Z*-value and *p*-value for testing the null hypothesis that the mean difference between groups is 0. The *Q*-statistic provides a test of the null hypothesis that all studies in the analysis share a common effect-size. The *I*^2^ statistic shows what proportion of the observed variance reflects differences in true effect-sizes rather than sampling-error. *T*^2^ is the variance of true effect-sizes or the between study variance. *T* is the standard deviation of true-effects (Borenstein et al., 2009).

### Data analysis

Meta-analysis was undertaken using Comprehensive Meta-Analysis Software Version 3 (CMA Version-3). The primary analysis compared the effect of intervention groups on depressive and anxiety symptoms and QoL scores. A funnel plot was used to investigate any publication bias. Sensitivity analyses were performed using “one-study-removed” analyses. A random-effects model was used in all analyses, weighting the studies based on the sample size/standard error. In cases when pre-post correlations were not reported in the published papers, we conducted sensitivity-analysis using a correlation of 0, 0.5, and 0.9, and found the results of our outcomes of interest to be the same, thus we used a 0 correlation for all analyses.

## Results

### Study selection

**Figure 1:**
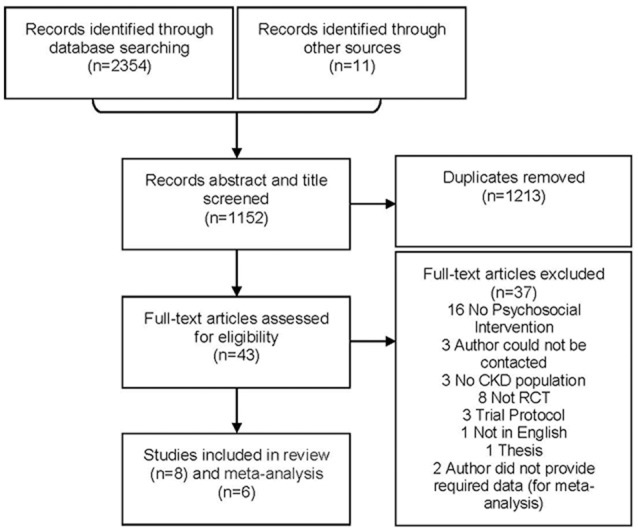
Flow Chart Showing the Retrieval Process of Trails included in the Meta-analysis.

Search of databases retrieved 2,365 papers with 1,213 duplicates, leaving 1,152 papers. Title/abstract screening excluded 1,109; thus, 43 remained for full-text review and ultimately eight were included (six in the meta-analysis). Initially 11 studies were identified, but three of these supplied insufficient detail to determine whether the interventions could be considered psychosocial or not and thus whether the studies met the inclusion criteria. The authors of these studies did not respond to requests for further information so the studies were not included in the systematic review or meta-analysis (Tsay and Hung, 2004; Tsay et al., 2005; Lii et al., 2007). Two additional studies did meet inclusion criteria for the systematic review, but were unable to provide requisite statistical information to be included in the meta-analysis: they were still included in the systematic review (Moattari et al., 2012; Hare et al., 2014). A PRISMA flow-diagram shows the selection of papers for inclusion and exclusion (Figure 1).

### Study characteristics

Study specifications are listed in Table 1. One study used a three-group, parallel-design, comparing a psychosocial intervention with supportive therapy, or usual care (UC) (Rodrigue et al., 2011). We extracted data only from the psychosocial intervention and UC groups. Two studies (Sharp et al., 2005; Hare et al., 2014) employed a deferred entry method and therefore only outcomes where the authors maintained a RCT design (before the wait-list group was assigned to the treatment group) were included in the meta-analysis. All included studies reported outcomes as pre and post means and SD within each group.

**Table 1:** Characteristics of included studies.

**References**	**Country**	**Study design**	**Participants**	**Intervention group**	**Comparison group**	**Time of assessment**	**Outcome measures**	**Differences between groups**	**Follow up**	**Intervention participation/adherence documented in study**	**Journal**
Chan et al., 2015	Hong Kong	Parallel group *M* ± *SD* completers only	Chronic kidney (creatinine clearance <15 mL/min) Patients Randomized *n* = 29)	Patients -enhanced psychosocial support (*n* = 14; M age 82 ± 4; Female = 6 [43%])	Patients—Usual care (*n* = 15[1 drop out]; M age 81 ± 6; Female = 8 [53%])	During intervention (4, 12 weeks), post intervention	Patients: MQOL, enhanced psychosocial support *n* = 4; Usual Care *n* = 5	Social support lowered perceived caregiver burden and caregiver anxiety at 4, 12 weeks.	None	Not addressed	Am J Kidney Dis
			Carers Randomized *n* = 29)	Carers—enhanced psychosocial support (*n* = 14; M age 64 ± 13; Female = 11 [79%])	Carers—usual care (*n* = 15[1 drop out]; M age 56 ± 13; Female = 11 [73%])		Carers: ZBI, HADS enhanced psychosocial support *n* = 4; Usual Care *n* = 5			Not addressed	
Cukor et al., 2014	USA	Cross over *M* ± *SD* completers only	Hemodialysis patients (Randomized *n* = 65)	CBT (*n* = 38; M age NP; Female = 27 [71%])	Waitlist control (usual care) (*n* = 27 M age NP; Female = 20 [73%])	Pre-post Intervention; follow up	BDI-II, HAM-D, KDQOL, SCID IDWG CBT *n* = 33; Usual Care *n* = 26	CBT decreased depressive symptoms, increased quality of life post intervention and at follow up.	None	Not addressed	J Am Soc Nephrol
Duarte et al., 2009	Brazil	Parallel group *M* ± *SD* completers only	Hemodialysis patients (Randomized *n* = 90)	CBT including training social abilities and assertiveness (*n* = 46; M age 52 ± 16; Female = 26 [63%])	Usual care (*n* = 44; M age 54 ± 13; Female = 24 [54%])	Pre-post Intervention; follow up	BDI, MINI, KDQOL CBT *n* = 41; Usual Care *n* = 44	CBT increased quality of life at post intervention and decreased depressive symptoms at post intervention and follow up.	6 months Intervention *n* = 36; Control *n* = 38	CBT participation 78.5%. UC Participation 85%.	Kidney Int
Hare et al., 2014^*^	UK	Deferred Entry ITT analysis	Peritoneal dialysis patients (Randomized *n* = 15)	Liquid Intake Programme^*^ (LIP) (*n* = 8; M age 60; Female = 0 [0%])	Deferred-entry group (usual care) (*n* = 7; M age 60; Female = 1 [7%])	Pre-post Intervention; follow up (post intervention assessments at 1 weeks post intervention completion)	HADS, SF-36, BP, IDWG LIP *n* = 8; Usual Care *n* = 7	LIP improved health status at 6 week follow up but not depressive symptoms or QoL. Longitudinal analysis showed the LIP decreased depressive symptoms	6 weeks Intervention *n* = 8; Control *n* = 7	Not addressed	Nephrol Dial Transplant
Moattari et al., 2012	Iran	Parallel group *M* ± *SD* completers only	Hemodialysis patients (Randomized *n* = 50)	Empowerment program (*n* = 25; M age 38 ± 11; Female = 10 [40%])	Usual care (*n* = 25; M age 37 ± 11; Female = 8 [30%])	Pre Intervention and follow up	QoL, SUPPH BP, IDWD, Na+, K+, Cr, BUN, P, Ca+, H&H	The empowerment program increased QoL, self-care self-efficacy, stabilized BP and increased H&H.	6 weeks Empowerment program *n* = 25; Usual Care *n* = 23	Not addressed	Health Qual Life Outcomes
Rodriguez Garcia and Rodriguez Garcia, 2011	USA	Parallel group ITT (maximum-likelihood estimates)	Adults approved for kidney transplantation (Randomized *n* = 65)	quality of life therapy (QOLT) (*n* = 22; M age 53 ± 11; Female = 10 [46%])	Usual care (*n* = 20; M age 52 ± 12; Female = 11 [55%]) supportive therapy (ST) (*n* = 20; M age 48 ± 11; Female = 12 [60%])	Pre, post Intervention, and follow up (post intervention assessments at 1 weeks post intervention completion)	QOLI, SF-36, POMS, HSCL, MSIS QOLT *n* = 20; Usual Care *n* = 20; ST *n* = 19	QOLT increased QoL at 1 and 12 w follow up. QOLT increased social intimacy and decreased social distress at 12 w follow up. QOLT and ST lowered psychological distress at 1w follow up.	12 weeks QOLT *n* = 17; Usual Care *n* = 18; ST *n* = 18	QOLT • full dose (*n* = 17) Partial dose (*n* = 5) ST—full dose (*n* = 17) Partial dose (*n* = 2)	Nephrol Dial Transplant
Sharp et al., 2005^*^	UK	Deferred Entry ITT (replace with group median)	Hemodialysis patients (Randomized *n* = 56)	Glasgow University Liquid-Intake Program (GULP)^*^ (*n* = 29; M age 56.05 ± 12.73; Female = 11 [38%])	Deferred-entry group (usual care) (*n* = 27 M age 52.52 ± 12.70; Female = 7 [26%])	Pre-post Intervention.	HADS, SF-36 IDWG GULP *n* = 29; Usual Care *n* = 27	GULP improved health status post intervention.	None	GULP— 100% dose (*n* = 17) 75%dose (*n* = 5) 50%dose (*n* = 2) 25%dose (*n* = 1)	Am J Kidney Dis
Song et al., 2009	USA	Parallel group *M* ± *SD* completers only	African American hemodialysis and peritoneal patients (Randomized *n* = 58)	SPIRIT patients (*n* = 29; M age 58.31 ± 11.8; Female = 10 [35%])	Usual Care patients (*n* = 29; M age 57.55 ± 12.2; Female = 15 [52%])	Pre Intervention and follow p	S-PRT SPIRIT Patients *n* = 29; Usual Care patients *n* = 29	SPIRIT did not improve QoL	12 weeks SPIRIT Patients *n* = 27; Usual Care patients *n* = 29	SPIRIT participation 100%	Res Nurs Health.
			Carers Randomized *n* = 58)	SPIRIT carers (*n* = 29; M age NP; Female = NP)	Usual Care carers (*n* = 29; M age NP; Female = NP)		SPIRIT carers *n* = 29; Usual Care carers *n* = 28		SPIRIT carers *n* = 27; Usual Care carers *n* = 27		

BDI, Becks Depression Inventory; BP, Blood Pressure; PAIS, Psychological Adjustment to illness Survey; BUN, Blood Urea Nitrogen; Ca+, Calcium; DCS, Decisional Conflict Scale; DSI, Dialysis Symptom Index; DMCS, Decision-Making Confidence Scale; GCD, Goals of Care document; HADS, Hospital Anxiety and Depression Scale; HAM-D, Hamilton Rating Scale for Depression; H&H, hemoglobin and haematocrit; IDWG, intradialytic weight gain; HSCL, Hopkins Symptom Checklist-25; K+, potassium; KDQOL, Kidney Disease Quality of Life Short Form; LSNS, Lubben Social Network Scale; PPS, Palliative Performance Scale; MINI, Major Depression module Mini International Neuropsychiatric Interview; MQOL, The McGill Quality of Life Questionnaire; MSIS, Miller Social Intimacy Scale; NP, Not provided; P, Phosphorous; POMS, Profile of Mood States; QoL, Ferrans and Powers Quality of Life Scale; QOLI, Quality of Life Inventory; SCID-I, Structured Clinical Interview for DSM Major Axis I Disorders; SCID-II, Structured Clinical Interview for DSM personality Disorders; SF, 36/12-Medical Outcomes Study Short Form; S-PRT-28, item Self-Perception and Relationship Tool; SUPPH, Strategies Used by People to Promote Health; Na, Sodium; ZBI, The Zarit Burden Interview. Underlined studies were not used in the meta-analysis.

* *Indicates that the same intervention was used*.

Sample sizes ranged from 15 to 90 and mean age ranged from 52 to 82 years. Two studies did not report mean participant age (Cukor et al., 2014; Chan et al., 2015). For one study, this information was provided by the author upon request (Chan et al., 2015). The percentage of women ranged from 0 to 79%. In all but three studies (Rodrigue et al., 2011; Chan et al., 2015) participants were undergoing haemodialysis and thus were in stage-5 of CKD, or ESKD, and were recruited from dialysis treatment centers or hospitals. In one study participants were undergoing peritoneal-dialysis (Hare et al., 2014). In another study, participants were waiting for kidney transplantation; 23% were not undergoing dialysis, 58% were undergoing haemodialysis, and 19% were undergoing peritoneal-dialysis (Rodrigue et al., 2011). In the third study, participants had selected not to undergo dialysis or enlist for kidney transplantation (Chan et al., 2015). The psychosocial interventions in each study varied in their components, frequency and length as reported in Table 2 (template for intervention description and replication [TIDiER] table).

**Table 2:** TIDiER table describing characteristics of the psychosocial interventions.

**References**	**Personnel delivering treatment (WHO provided)**	**Setting (WHERE)**	**Psychological component (WHY/WHAT)**	**Social support component (WHY/WHAT)**	**Topics addressed/components (WHY/WHAT)**	**Individual/Group (HOW)**	**Mode of delivery (HOW)**	**Invention duration (WEND and HOW MUCH)**
Chan et al., 2015	Palliative care nurse, social worker, and physician	Renal palliative clinic	Education and social support	Social isolation addressed	“*Palliative care nurse:* education about chronic kidney failure and related problems; patient medical care (drug, diet adherence); patient and carer psychological aspect recommendations; management of the patient's symptoms and skills in coping with them. Problem-solving intervention. Symptom advice, monitored adherence treatments and fluid recommendations. *Social worker:* assessment of social support and caring issues; orientation in stress management; improvement of communication skills in family; orientation of caregivers to relaxation methods; interventions as needed, community service referral, coping skill training, respite care for intervention—social support and advice concerning financial issues and difficulties in placing the patient in home care and arranging respite care for caregivers (Chan et al., 2015)”	Individual	In person	6 months: 30 m/1–2 month
Cukor et al., 2014	Psychologist	Two outpatient hemodialysis centers	Cognitive Behavioral Therapy	Social isolation addressed	“Education about depression and medical illnesses, medication adherence, increasing enjoyable activities, addressing cognitive distortions, increasing positive social contacts—initiating contact, building support network (Cukor et al., 2014)”	Individual	In person	3 months: 60 m/week (10 sessions total)
Duarte et al., 2009	Psychologist	Two outpatient hemodialysis centers	Cognitive Behavioral Therapy	Training on social abilities and assertiveness	“Education of renal disease, dialysis, depression, self-monitoring, cognitive restructuring, programming pleasurable activities, social abilities and assertiveness, relaxation (Duarte et al., 2009)”	Group	In person	3 months: 90 m/week
Hare et al., 2014^*^	Trainee Psychologist	Renal Service Home Therapies Department	Cognitive Behavioral Therapy	Maximizing social support for the benefit of fluid adherence	“The content of the intervention utilized CBT techniques, encompassing educational, cognitive and behavioral components; aimed to assist patients' self-management of fluid. Participants were provided with a structured LIP treatment manual; including record sheets, goal-setting sheets and daily planners for fluid intake and a relaxation CD. In accordance with CBT principles, participants were encouraged to complete homework between sessions; to maximize learning in everyday life (Hare et al., 2014)”	Group	In person	4 weeks: 1 h/week
Moattari et al., 2012	Psychiatric nurse and the second author	Hemodialysis Center	Individual and group counseling	Education is relationships with family and friends	“Individual counseling sessions were conducted by a psychiatric nurse and focused on stress management, problem-focused and emotion-focused coping strategies, social support and motivation A behavior change plan was based on the patient's priority. Self-efficacy in regards to each behavior change plan was assessed by a visual analog scale. Patients' families were involved in the process of empowerment at the patient's request. Patients were informed about available social support and were referred to the appropriate centers and experts if necessary (Moattari et al., 2012)”	Individual and Group	In person	6 weeks: 90–120 m/week
Rodriguez Garcia and Rodriguez Garcia, 2011	Psychologists and social workers	Teaching Hospital	Individual counseling	Improving family relationships	“Identifying contributors to QOL, and problem solving, to improve QOL, The desire of patients to improve their satisfaction with the quality of their relationships with family members or friends was common. This involved assessing the relationship, understanding their thoughts and feelings about the relationship, articulating how they want the relationship to change/be different, and setting goals for the relationship (Rodrigue et al., 2011)”	Individual	In person	2 months: 50 m/week
Sharp et al., 2005^*^	Supervised trainee clinical psychologist	Outpatient hemodialysis units	Cognitive Behavioral Therapy	Maximizing social support for the benefit of fluid adherence.	“Identification of associations between thoughts, emotions, and behaviors, rationality and accuracy of their beliefs in an attempt to modify thoughts identified as maladaptive, relaxation techniques. Discussing the importance of effective social support networks. Suggestions given on how to interact appropriately with others regarding the management of fluid consumption and gain optimal social support from significant others (Sharp et al., 2005)”	Group	In person	4 weeks: 60 m/week
Song et al., 2009	Medical nurse	Outpatient dialysis units	Roleplaying, skills demonstration, counseling	Improving dyad relationship	“Describing illness representations to achieve a deeper understanding of patient's illness experience and the carers experience. Identifying and exploring gaps and concerns the dyad may have regarding illness progression, life sustaining treatment and decision making. Sharing of views and ideas about death and dying and end-of-life care. Encouraging the patient to clarify goals of care and express concerns. Assessment of additional support needs (Song et al., 2009)”	Dyad	In person	Single Session: 60 m

Three studies used CBT (Sharp et al., 2005; Duarte et al., 2009; Cukor et al., 2014). One study used individual counseling (Rodrigue et al., 2011), one used individual education and social support (Chan et al., 2015), and one used a combination of counseling and roleplaying skills demonstration delivered in the dyad (Song et al., 2009). Social isolation was addressed by two studies (Cukor et al., 2014; Chan et al., 2015). One study aimed to train participants on social abilities and assertiveness (Duarte et al., 2009) another to improve family relationships (Rodrigue et al., 2011), another to improve the dyad relationship (Song et al., 2009) and one study aimed to maximize social support for the benefit of fluid adherence (Sharp et al., 2005). Three studies delivered the interventions individually (Rodrigue et al., 2011; Cukor et al., 2014; Chan et al., 2015) and two studies delivered the intervention in a group setting (Sharp et al., 2005; Duarte et al., 2009) and one study delivered the intervention in a dyad (Song et al., 2009). In four of the included trials, the intervention was delivered by a psychologist (Duarte et al., 2009; Cukor et al., 2014) a supervised trainee psychologist (Sharp et al., 2005) or a psychologist in conjunction with social workers (Rodrigue et al., 2011). One study employed a combination of a palliative care nurse, social worker, and physician to deliver the intervention (Chan et al., 2015) and in one study the intervention was delivered by a medical nurse (Song et al., 2009). Underlined studies were not used in the meta-analysis.

* *Indicates that the same intervention was used*.

### Risk of bias within studies and grades of recommendation, assessment, development, and evaluation

All authors were contacted to request any additional unpublished data. Two authors responded to confirm that they had no unpublished data (Sharp et al., 2005; Chan et al., 2015). As can be seen in Table 3, on each of the domains the vast majority of the included RCTs were rated as having a low or unclear risk of bias, which is insufficient to justify downgrading the level of evidence. However, as seen in the meta-analysis results (below), heterogeneity exists between study outcomes for depressive and anxiety symptoms. This heterogeneity appears to result from differences in measurement tools and populations studied. In terms of PICOs, we consider the population, interventions, comparison, and outcomes to be sufficiently direct to address the question at hand. In terms of precision, we consider the sample sizes to be sufficiently large for the depressive symptoms and QoL outcomes, and the CI's on these outcomes to be sufficiently narrow. For anxiety symptoms, the total sample was only *n* = 197. Finally, in terms of publication bias, funnel plots did not appear to be asymmetric for depressive symptoms or QoL outcomes. There were too few studies of anxiety symptoms to assess funnel plots for this outcome reliably. Given the above considerations, we suggest that the GRADE of evidence should be downgraded to moderate from high for depressive symptoms outcomes and from high too low for anxiety symptoms outcomes.

**Table 3:** Risk of bias assessment for included studies.

**References**	**Random sequence generation**	**Allocation concealment**	**Blinding of outcome assessment**	**Attrition bias**	**Selective reporting**	**Other bias**
Chan et al., 2015	Low	Low	Low	High	Low	UC
Cukor et al., 2014	UC	UC	Low	Low	UC	Low
Hare et al., 2014^*^	Low	UC	High	Low	UC	Low
Moattari et al., 2012	UC	UC	Low	Low	UC	Low
Duarte et al., 2009	Low	Low	Low	Low	UC	UC
Rodriguez Garcia and Rodriguez Garcia, 2011	UC	UC	Low	Low (ITT)	UC	Low
Sharp et al., 2005^*^	Low	Low	High	Low (ITT)	UC	UC
Song et al., 2009	Low	Low	UC	Low	UC	UC

UC, Unclear; ITT, Intention to treat; Random sequence generation' “UC” method of randomization not specified; Allocation concealment: “UC” studies did not report if allocation concealment was maintained; Blinding of outcome assessment: “UC” studies did not report if assessors were blind, in “High” studies assessor were not blind; Attrition bias: in the “High” study 10/14 in the intervention group and 10/15 in the UC condition passed away before study completion; Selective reporting: “UC” Protocols were not available; other sources of bias: “UC” gender imbalance in the study groups (Sharp et al., 2005). Baseline differences present between groups (Duarte et al., 2009; Song et al., 2009). In the study by Chan et al., (Chan et al., 2015) it was not stated why Mann-Whitney test were used instead of T-tests, assumption testing was not reported (Chan et al., 2015). Underlined studies were not used in the meta-analysis.

* *Indicates that the same intervention was used*.

### Limitations in generalizability and in information reported in included studies

The authors did not use a clinical cut-off score of depressive and anxiety symptoms as an inclusion criterion in five studies that measured depressive or anxiety symptoms as an outcome (Sharp et al., 2005; Rodrigue et al., 2011; Chan et al., 2015). Neither did these authors report the percentage of participants with a clinical level of depressive and anxiety symptoms at baseline. In one study, it was not stated whether informed consent was obtained from participants (Sharp et al., 2005). Implications for policy were not addressed in four studies (Sharp et al., 2005; Duarte et al., 2009; Song et al., 2009; Cukor et al., 2014; Chan et al., 2015). One study was underpowered (Chan et al., 2015) and another study included only patients of African American ethnicity (Song et al., 2009). One study had a gender imbalance in the carer study group (76% female; Chan et al., 2015) another had a primarily male sample (Hare et al., 2014). One study had non-significant differences in groups' baseline Beck Depression Inventory scores (Duarte et al., 2009).

### Meta-analysis

Table 4 presents a list of studies and tools used to examine depressive symptoms, anxiety symptoms and QoL, at post intervention and at 3 months post intervention completion. Table 4 shows when a composite mean has been used and from which tools this mean is has been derived, as indicated in parenthesis.

**Table 4:** List of studies and tools used in meta-analysis to examine depression, anxiety, or quality of life.

		**Depression**	**Anxiety**	**Quality of Life**	
**SCALES USED**					
Study	Sample	Post Intervention	Post Intervention	Post Intervention	3 months follow up from intervention completion
Chan et al., 2015	Patient			MQOL	
	Carer	HADS	HADS		
Cukor et al., 2014	Patient	BDI, HAM-D (Composite score of these used)		KDQOL-SF	
Duarte et al., 2009	Patient	BDI, MINI (Composite score of these used)		KDQOL-SF	
Rodriguez Garcia and Rodriguez Garcia, 2011	Patient	HSCL, POMS, mentally unhealthy days (Composite score of these used)	HSCL, POMS and mentally unhealthy days	SF-36 (2 composite scales) QOLI (Composite score of these used)	SF-36 (2 composite scales) QOLI (Composite score of these used)
Sharp et al., 2005	Patient	HADS	HADS	SF-36 (8 subscales)	
Song et al., 2009	Patient			S-PRT	S-PRT
	Carer			S-PRT	S-PRT

*S-PRT, 8-item Self-Perception and Relationship Tool; BDI, Becks Depression Inventory; HAM-D, Hamilton Rating Scale for Depression; HSCL, Hopkins Symptom Checklist-25; KDQOL, Kidney Disease Quality of Life Short Form; MINI, Major Depression module Mini International Neuropsychiatric Interview; POMS, Profile of Mood States; HADS, The Hospital Anxiety and Depression Scale; MQOL, The McGill Quality of Life Questionnaire; and QOLI, the Quality of Life Inventory*.

#### Depressive symptoms outcomes

**Figure 2:**
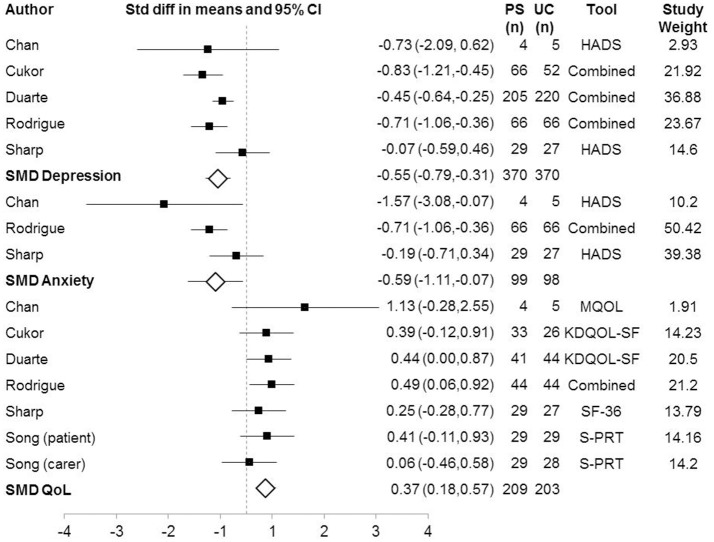
Forest Plot of Psychosocial Interventions on Depressive and Anxious Symptoms by Study. Study used a combination of measured to measure the outcome of interest = Combined; Standardized mean difference = SMD.

At post-intervention, five studies measured depressive symptoms (*n* = 740). Four of these studies measured symptoms in patients (Sharp et al., 2005; Duarte et al., 2009; Rodrigue et al., 2011; Cukor et al., 2014) and one in carers (Chan et al., 2015) (Figure 2). The results indicate a medium effect (g) of the psychosocial intervention compared to UC [*Z* = −4.467, *p* = < 0.001, *Q* = 7.266 (4 *df*), *I*^2^ = 44.950%, *T*^2^ = 0.031, *T* = 0.175]. Removal of any one study did not alter the results significantly. Subgroup analysis showed that in the three studies using a composite measure of depressive symptoms (see Table 4), that the *SMD* was −0.618, *CI* = −0.864 to −0.372, *p* = < 0.001. The *Q*-value was 4.040 (2 *df*), indicating that the effect-size still varied across these studies, likely due to differences in the measurement tools. Conversely, for the two studies that used the Hospital Anxiety and Depression Scale (HADS) the effect-size was not significant (*SMD* = −0.154, *CI* = −0.643 to 0.335, *p* = 0.538), therefore the heterogeneity between studies appears to result from using different tools to measure depressive symptoms. Funnel plot results are included in the Appendix. These results indicate that psychosocial interventions offer greater relief of depressive symptoms than usual care in patients with CKD.

#### Anxiety outcomes

The analysis of anxiety symptoms post-intervention included three studies (Figure 2). One of these studies measured symptoms in carers (Chan et al., 2015) and two measured symptoms in patients (Sharp et al., 2005; Rodrigue et al., 2011) (*n* = 197). The results indicated a medium effect of the intervention [Z = −2.217, *p* = 0.027, *Q* = 4.362 (2 df), *I*^2^ = 54.153%, *T*^2^ = 0.108, *T* = 0.329]. Two studies are largely responsible for the findings, as confirmed by one study removed analysis, which showed that absence of either of these studies resulted in a non-significant difference between intervention and control groups: (Chan et al., 2015; *SMD* = −0.485, *CI* = −0.992 to 0.023, *Z* = −1.870, *p* = 0.061; Rodriguez Garcia and Rodriguez Garcia, 2011; *SMD* = −0.693, *CI* = −2.004 to 0.617, *Z* = −1.037, *p* = 0.300). The included studies are heterogeneous as one measured anxiety in carers, another anxiety in patients and the third used a combined measure of depressive and anxiety symptoms in patients. Therefore, the heterogeneity between studies appears to result from including different population groups. We performed subgroup analyses comparing outcomes using the different depressive symptom measurement tools. This showed that the two studies that used the HADS did not find a significant effect of psychosocial interventions on anxiety outcomes, *SMD* = −0.693, *CI* = −2.004 to 0.617, *Z* = −1.037, *p* = 0.300, *Q* = 2.922(1 *df*), *I*^2^ = 65.778%, *T*^2^ = 0.634, *T* = 0.796.

#### Quality of life outcomes

The analysis of QoL symptoms post-intervention included six studies (*n* = 412). Five study measured QoL in patients (Sharp et al., 2005; Duarte et al., 2009; Rodrigue et al., 2011; Cukor et al., 2014; Chan et al., 2015). In one study, QoL was measured in both patients and carers (Song et al., 2009), therefore this study was entered twice into the analysis. The results indicated a small effect of the intervention (*Z* = 3.734, *p* = < 0.001, *Q* = 3.096(6 df), *I*^2^, *T*^2^, *T* = 0). One-study-removed analysis showed that removal of any one study did not change the overall result. Two studies assessed QoL at 3-months follow up and thus a separate meta-analysis was conducted including only these studies (Song et al., 2009; Rodrigue et al., 2011). This meta-analysis indicated no sustained effect of the intervention at 3 months (*SMD* = 0.256, *CI* = −0.054 to 0.567, *Z* = 1.618, *p* = 0.106, *Q* = 2.330(2 *df*), *I*^2^ = 14.170%, *T*^2^ = 0.011, *T* = 0.104).

## Discussion

In this review, psychosocial interventions appeared to reduce depressive symptoms and anxiety and improved QoL outcomes in patients with CKD and/or their carers, compared to usual care (UC). For depressive symptoms, the magnitude of the *SMD* reflects a medium benefit of the intervention; however the effect-size varied across studies. Three studies used a combined measure of depressive symptoms and found a significant effect, while two used the HADS and found no significant effect, suggesting heterogeneity resulting from differences in outcome measures. Thus, we suggest that the level of evidence for psychosocial interventions compared to usual care on depressive symptoms should be interpreted as moderate rather than high.

For anxiety, the magnitude of the *SMD* similarly suggests a medium benefit of psychosocial interventions; however, this is based on only three studies and did not withstand sensitivity analysis. Subgroup analysis indicated heterogeneity across studies, which again was associated with different assessment tools, as well as differences in the populations studied. The two studies involving patients contributed 90% to the finding (Sharp et al., 2005), leaving the effect of psychosocial interventions on carers' anxiety, unresolved. For QoL outcomes, the magnitude of the *SMD* suggests a small benefit for the intervention; however, these same benefits were not seen at 3 months follow-up.

Carers and patients were analyzed together in the current study, raising the question of whether these groups are sufficiently comparable to be analyzed together. Removal of the studies involving carers did not change the overall result of the meta-analysis, indicating that outcomes were similar across the two population groups in our study. This is particularly relevant for the QoL outcome, as one study measured QoL in both patients and carers (Song et al., 2009) and was entered twice into the analysis. The outcome of these two populations is likely correlated, so we conducted a separate meta-analysis including only studies involving patients, which similarly showed that psychosocial interventions improved QoL compared to UC.

These findings are consistent with our previous meta-analysis indicating that psychosocial interventions reduce depressive symptoms and anxiety in patients with cardiovascular disease (Ski et al., 2015), though this was—like the present study—limited by only five studies being identified for inclusion (Ski et al., 2015). The small number of identified studies is testament to the need for further research in this field. Few researchers are currently using psychosocial interventions in the health research field, perhaps due to a lack of clear definition regarding what constitutes a psychosocial intervention. For example, in a meta-analysis of 44 trials involving older healthy adults or adults with sub-clinical depression, the authors concluded that psychosocial interventions improved QoL and reduced depressive symptoms (Forsman et al., 2011a,b). However, a range of interventions were classed as psychosocial, including exercise and reminiscence trials (Forsman et al., 2011a,b). Another meta-analysis of RCTs of psychosocial interventions compared to UC in family members and patients with various chronic illnesses and reported that psychosocial interventions had a small significant positive effect on depressive symptoms (Martire et al., 2004). However, the characteristics of the interventions of the included studies were not well-described and the authors stated that they included all “nonmedical interventions that are psychologically, socially, or behaviorally oriented” (Martire et al., 2004). Therefore, previous meta-analyses have included studies that would not be deemed psychosocial interventions according to our definition (Thompson and Ski, 2013).

This prompts a discussion about what constitutes a psychosocial intervention. As highlighted in the meta-analyses mentioned earlier (Martire et al., 2004; Thompson and Ski, 2013) the term “psychosocial” is often used in the literature to describe an intervention that would more accurately be described as behavioral, educational, psychological, or social. We suggest that in order to be considered psychosocial, an intervention must combine a clearly defined psychological component with a social component (Thompson and Ski, 2013). Better reporting of the intervention characteristics would aid in transparency regarding whether interventions are psychosocial or not. At present, there is a lack of consistency in how psychosocial interventions are defined, delivered and tested, and this makes the evaluation of the efficacy of such interventions complicated (Thompson and Ski, 2013). Accordingly, we took care to ensure that all of the primary studies included in the present study complied with the suggested definition of psychosocial interventions i.e., they combined psychological and social components.

There are three main limitations to the current meta-analysis. Firstly, all of the primary studies have small sample sizes. Secondly, three of the primary studies have no assessment of depressive symptoms at follow-up, two have no assessment of anxiety outcome at follow-up and five have no assessment of QoL outcomes at follow-up. Additionally, two studies were identified in the literature but could not be included in the meta-analysis. One of these studies failed to find a beneficial effect of the psychosocial intervention (Hare et al., 2014), while the other found a beneficial effect on QoL outcomes (Moattari et al., 2012).

As highlighted in the results, in five studies the authors did not use a clinical cut-off score of depressive and anxiety symptoms as an inclusion criterion, but measured depressive or anxiety symptoms as an outcome. These authors also did not report the percentage of participants with clinical levels of depression and/or anxiety at baseline (Sharp et al., 2005; Rodrigue et al., 2011; Hare et al., 2014; Chan et al., 2015). A reduction in depressive symptoms and anxiety would be difficult to achieve if a number participants experienced only a low level of depressive and anxiety symptoms at baseline. Therefore, it is not surprising that three studies which did not use a clinical cut-off score of depressive symptoms and anxiety at baseline (Sharp et al., 2005; Hare et al., 2014; Chan et al., 2015), failed to find a reduction in these post-intervention. In future studies, we would suggest that psychosocial interventions aimed to decrease depressive and anxiety symptoms be targeted toward those experiencing such symptoms at baseline, in order to best serve the most relevant patient populations.

None of the included studies reported data on patient consent rates and uptake of the intervention. Only three studies reported patient adherence to the intervention (Sharp et al., 2005; Duarte et al., 2009; Rodrigue et al., 2011) as reported in Table 1. Given the time requirements of dialysis treatment on CKD patients and the extensive contact they have with the medical system, many patients may be reluctant to participate in a time intensive psychosocial intervention. This is a particular concern given that many patients feel tired and weak after dialysis. This is an important consideration in terms of clinical practice, as not only should clinical interventions be effective, but they must also be feasible and acceptable to the patients, in order to achieve sustainable implementation within clinical settings.

Finally, CKD and dialysis treatment is associated with a number of symptoms which mirror those of anxiety and depression (American Psychiatric Association, 2013), such as sleeping problems, changes in appetite, fatigue, and changes in cognition (Kidney Health Australia, 2017; NHS, 2017). This is problematic in terms of self-report measures of depressive and anxiety symptoms, as these may capture symptoms of the disease and dialysis treatment, rather than being an indicator of the experience of depressive and anxiety symptoms. Therefore, careful consideration should be given to the tools used in order to accurately measure depressive and anxiety symptoms in populations with CKD.

Overall, the results of the current meta-analysis indicate that various psychosocial interventions may reduce depressive symptoms and improve QoL in the carers of and patients diagnosed with CKD. Preliminary evidence suggests that there may be a benefit of psychosocial interventions on anxiety symptoms, for patients diagnosed with CKD.

## Author contributions

MP conceived the study including data sources and search strategy, conducted the systematic search, performed study selection, extracted data, performed data synthesis, and wrote the manuscript. DT conceived the study including data sources and search strategy and critically appraised the manuscript. DC conceived the study. SM performed study selection and extracted data. CS conceived the study including data sources and search strategy and critically appraised the manuscript. All authors take responsibility for the contents of this article.

### Conflict of interest statement

The authors declare that the research was conducted in the absence of any commercial or financial relationships that could be construed as a potential conflict of interest.
