# Supplementary material for: Psychosocial Interventions for Depressive and Anxiety Symptoms in Individuals with Chronic Kidney Disease: Systematic Review and Meta-Analysis
Source: Front Psychol. 2017 Jun 13;8:992. doi: 10.3389/fpsyg.2017.00992 (PMC5468538; doi:10.3389/fpsyg.2017.00992)
Supplement: Supplementary file 1 [file DataSheet1.docx]

**Appendix**

Exact search strategy for each database

PubMed

Specific title or MeSH words, ‘kidney-disease,’ or ‘renal-disease,’ or  ‘renal-insufficiency,’ or ‘dialysis,’ or ‘peritoneal-dialysis,’ or ‘hemodialysis,’ or ‘haemodialysis,’ or ‘kidney-function,’ or ‘kidney-failure,’ and the specific abstract words, ‘depression,’ or ‘depressive,’ ‘depressed,’ or ‘melancholia,’ or ‘dysthymia,’ or ‘mood,’ or ‘anxiety,’ or ‘anxious,’ or ‘quality-of-life,’ or ‘coping,’ or ‘stress,’ and the specific abstract word, ‘psych*,’ or ‘motivational-interviewing,’ or ‘motivational-behaviour,’ or ‘behaviour-interviewing,’ or ‘behaviour-change,’ or ‘motivational-behavior,’ or ‘behavior-interviewing,’ or ‘behavior-change,’ or ‘motivational-change,’ or ‘non-invasive-change.’

MEDLINE

Databases were searched for articles with no year restriction and containing the specific title or MeSH words, ‘kidney-disease,’ or ‘renal-disease,’ or  ‘renal-insufficiency,’ or ‘dialysis,’ or ‘peritoneal-dialysis,’ or ‘hemodialysis,’ or ‘haemodialysis,’ or ‘kidney-function,’ or ‘kidney-failure,’ and the specific abstract words, ‘depression,’ or ‘depressive,’ ‘depressed,’ or ‘melancholia,’ or ‘dysthymia,’ or ‘mood,’ or ‘anxiety,’ or ‘anxious,’ or ‘quality-of-life,’ or ‘coping,’ or ‘stress,’ and the specific abstract word, ‘psych*,’ or ‘motivational-interviewing,’ or ‘motivational-behaviour,’ or ‘behaviour-interviewing,’ or ‘behaviour-change,’ or ‘motivational-behavior,’ or ‘behavior-interviewing,’ or ‘behavior-change,’ or ‘motivational-change,’ or ‘non-invasive-change.’

CINAHL

Databases were searched for articles with no year restriction and containing the specific title words, ‘kidney-disease,’ or ‘renal-disease,’ or  ‘renal-insufficiency,’ or ‘dialysis,’ or ‘peritoneal-dialysis,’ or ‘hemodialysis,’ or ‘haemodialysis,’ or ‘kidney-function,’ or ‘kidney-failure,’ and the specific abstract words, ‘depression,’ or ‘depressive,’ ‘depressed,’ or ‘melancholia,’ or ‘dysthymia,’ or ‘mood,’ or ‘anxiety,’ or ‘anxious,’ or ‘quality-of-life,’ or ‘coping,’ or ‘stress,’ and the specific abstract word, ‘psych*,’ or ‘motivational-interviewing,’ or ‘motivational-behaviour,’ or ‘behaviour-interviewing,’ or ‘behaviour-change,’ or ‘motivational-behavior,’ or ‘behavior-interviewing,’ or ‘behavior-change,’ or ‘motivational-change,’ or ‘non-invasive-change.’

PsycINFO

Databases were searched for articles with no year restriction and containing the specific title words, ‘kidney-disease,’ or ‘renal-disease,’ or  ‘renal-insufficiency,’ or ‘dialysis,’ or ‘peritoneal-dialysis,’ or ‘hemodialysis,’ or ‘haemodialysis,’ or ‘kidney-function,’ or ‘kidney-failure,’ and the specific abstract words, ‘depression,’ or ‘depressive,’ ‘depressed,’ or ‘melancholia,’ or ‘dysthymia,’ or ‘mood,’ or ‘anxiety,’ or ‘anxious,’ or ‘quality-of-life,’ or ‘coping,’ or ‘stress,’ and the specific abstract word, ‘psych*,’ or ‘motivational-interviewing,’ or ‘motivational-behaviour,’ or ‘behaviour-interviewing,’ or ‘behaviour-change,’ or ‘motivational-behavior,’ or ‘behavior-interviewing,’ or ‘behavior-change,’ or ‘motivational-change,’ or ‘non-invasive-change.’

Scopus

Databases were searched for articles with no year restriction and containing the specific title words, ‘kidney-disease,’ or ‘renal-disease,’ or  ‘renal-insufficiency,’ or ‘dialysis,’ or ‘peritoneal-dialysis,’ or ‘hemodialysis,’ or ‘haemodialysis,’ or ‘kidney-function,’ or ‘kidney-failure,’ and the specific abstract words, ‘depression,’ or ‘depressive,’ ‘depressed,’ or ‘melancholia,’ or ‘dysthymia,’ or ‘mood,’ or ‘anxiety,’ or ‘anxious,’ or ‘quality-of-life,’ or ‘coping,’ or ‘stress,’ and the specific abstract word, ‘psych*,’ or ‘motivational-interviewing,’ or ‘motivational-behaviour,’ or ‘behaviour-interviewing,’ or ‘behaviour-change,’ or ‘motivational-behavior,’ or ‘behavior-interviewing,’ or ‘behavior-change,’ or ‘motivational-change,’ or ‘non-invasive-change.’

Web-of-Science

Databases were searched for articles with no year restriction and containing the specific title words, ‘kidney-disease,’ or ‘renal-disease,’ or  ‘renal-insufficiency,’ or ‘dialysis,’ or ‘peritoneal-dialysis,’ or ‘hemodialysis,’ or ‘haemodialysis,’ or ‘kidney-function,’ or ‘kidney-failure,’ and the specific abstract words, ‘depression,’ or ‘depressive,’ ‘depressed,’ or ‘melancholia,’ or ‘dysthymia,’ or ‘mood,’ or ‘anxiety,’ or ‘anxious,’ or ‘quality-of-life,’ or ‘coping,’ or ‘stress,’ and the specific abstract word, ‘psych*,’ or ‘motivational-interviewing,’ or ‘motivational-behaviour,’ or ‘behaviour-interviewing,’ or ‘behaviour-change,’ or ‘motivational-behavior,’ or ‘behavior-interviewing,’ or ‘behavior-change,’ or ‘motivational-change,’ or ‘non-invasive-change.’

SocIndex

Databases were searched for articles with no year restriction and containing the specific title words, ‘kidney-disease,’ or ‘renal-disease,’ or  ‘renal-insufficiency,’ or ‘dialysis,’ or ‘peritoneal-dialysis,’ or ‘hemodialysis,’ or ‘haemodialysis,’ or ‘kidney-function,’ or ‘kidney-failure,’ and the specific abstract words, ‘depression,’ or ‘depressive,’ ‘depressed,’ or ‘melancholia,’ or ‘dysthymia,’ or ‘mood,’ or ‘anxiety,’ or ‘anxious,’ or ‘quality-of-life,’ or ‘coping,’ or ‘stress,’ and the specific abstract word, ‘psych*,’ or ‘motivational-interviewing,’ or ‘motivational-behaviour,’ or ‘behaviour-interviewing,’ or ‘behaviour-change,’ or ‘motivational-behavior,’ or ‘behavior-interviewing,’ or ‘behavior-change,’ or ‘motivational-change,’ or ‘non-invasive-change.’

Cochrane Central Register of Controlled Trials (CENTRAL)

Specific title words, ‘kidney-disease,’ or ‘renal-disease,’ or  ‘renal-insufficiency,’ or ‘dialysis,’ or ‘peritoneal-dialysis,’ or ‘hemodialysis,’ or ‘haemodialysis,’ or ‘kidney-function,’ or ‘kidney-failure,’ and the specific abstract words, ‘depression,’ or ‘depressive,’ ‘depressed,’ or ‘melancholia,’ or ‘dysthymia,’ or ‘mood,’ or ‘anxiety,’ or ‘anxious,’ or ‘quality-of-life,’ or ‘coping,’ or ‘stress,’ and the specific abstract word, ‘psych*,’ or ‘motivational-interviewing,’ or ‘motivational-behaviour,’ or ‘behaviour-interviewing,’ or ‘behaviour-change,’ or ‘motivational-behavior,’ or ‘behavior-interviewing,’ or ‘behavior-change,’ or ‘motivational-change,’ or ‘non-invasive-change.’

Specific MeSH words, ‘kidney-disease,’ and ‘depression’, or ‘anxiety,’ and the specific title/abstract/keyword, ‘psych*,’ or ‘motivational-interviewing,’ or ‘motivational-behaviour,’ or ‘behaviour-interviewing,’ or ‘behaviour-change,’ or ‘motivational-behavior,’ or ‘behavior-interviewing,’ or ‘behavior-change,’ or ‘motivational-change,’ or ‘non-invasive-change.’

Funnel Plots

Depression


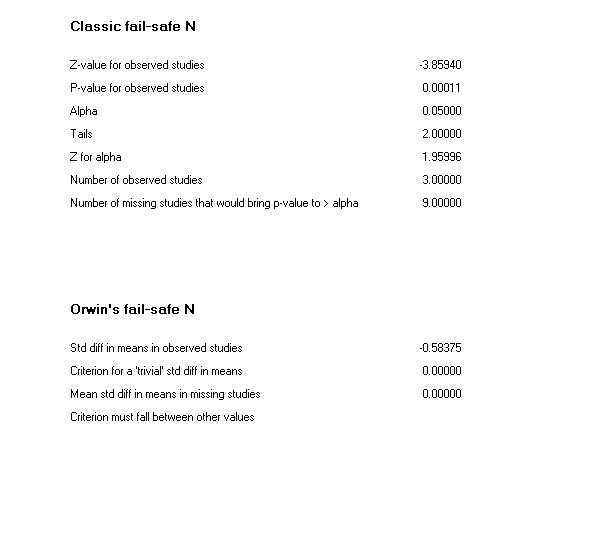


**0.2**

**0.4**

**0.6**

**0.8**

**-2.0**

**-1.5**

**-1.0**

**-0.5**

**0.0**

**0.5**

**1.0**

**1.5**

**2.0**

**Stddiff inmeans**

Anxiety


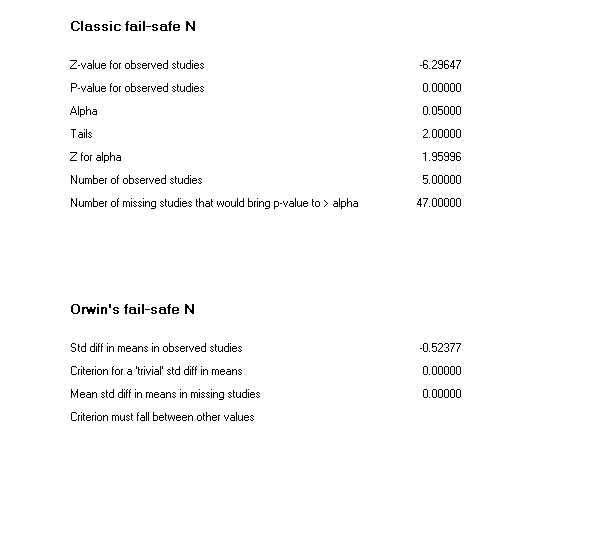


**Funnel Plot of Standard Error by Std diff in means**

**0.0**

**0.2**

**0.4**

**0.6**

**0.8**

**-2.0**

**-1.5**

**-1.0**

**-0.5**

**0.0**

**0.5**

**1.0**

**1.5**

**2.0**

**Stddiff inmeans**

Quality of Life


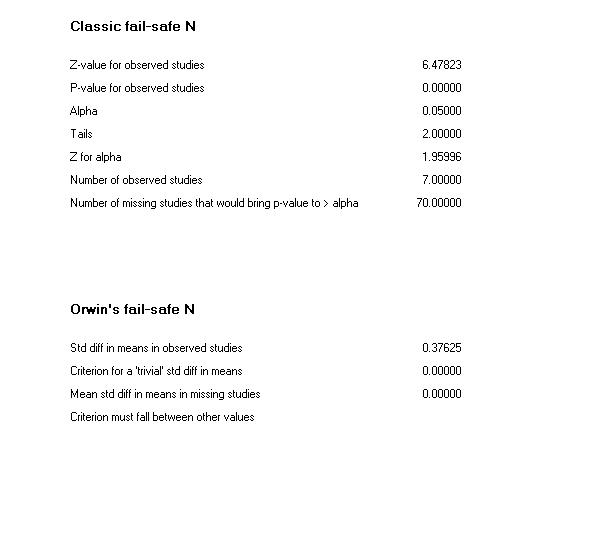


Quality of life – Patients only


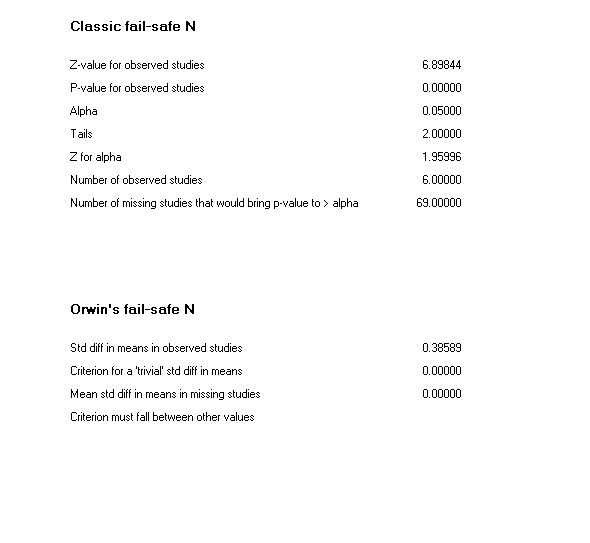


**0.2**

**0.4**

**0.6**

**0.8**

**-2.0**

**-1.5**

**-1.0**

**-0.5**

**0.0**

**0.5**

**1.0**

**1.5**

**2.0**

**Stddiff inmeans**
